# Artificial Intelligence in the Organization of Nursing Care: A Scoping Review

**DOI:** 10.3390/nursrep14040202

**Published:** 2024-10-02

**Authors:** João Ventura-Silva, Maria Manuela Martins, Letícia de Lima Trindade, Ana da Conceição Alves Faria, Soraia Pereira, Samuel Spiegelberg Zuge, Olga Maria Pimenta Lopes Ribeiro

**Affiliations:** 1 Abel Salazar Institute of Biomedical Sciences, 4050-313 Porto, Portugal; mmmartins@icbas.up.pt (M.M.M.); acafaria@ulsmave.min-saude.pt (A.d.C.A.F.); soraia.pereira@essnortecvp.pt (S.P.); 2 Northern Health School of the Portuguese Red Cross, 3720-126 Oliveira de Azeméis, Portugal; 3 CINTESIS@RISE, 4200-450 Porto, Portugal; olgaribeiro@esenf.pt; 4 Department of Nursing, Community University of the Chapecó Region (Unochapecó), Chapecó 89809-900, Brazil; leticia.trindade@udesc.br (L.d.L.T.); samuel.zuge@unochapeco.edu.br (S.S.Z.); 5 Grouping of Health Centers Ave/Famalicão, 4760-412 Vila Nova de Famalicão, Portugal; 6 Nursing School of Porto (ESEP), 4200-072 Porto, Portugal

**Keywords:** artificial intelligence, nurse administration, nursing, patient care management, organization and administration

## Abstract

Background: The integration of artificial intelligence (AI) in the organization of nursing care has continually evolved, driven by the need for innovative solutions to ensure quality of care. The aim is to synthesize the evidence on the use of artificial intelligence in the organization of nursing care. Methods: A scoping review was carried out based on the Joanna Briggs Institute methodology, following the PRISMA-ScR guidelines, in the MEDLINE, CINAHL Complete, Business Source Ultimate and Scopus^®^ databases. We used ProQuest—Dissertations and Theses to search gray literature. Results: Ten studies were evaluated, identifying AI-mediated tools used in the organization of nursing care, and synthesized into three tool models, namely monitoring and prediction, decision support, and interaction and communication technologies. The contributions of using these tools in the organization of nursing care include improvements in operational efficiency, decision support and diagnostic accuracy, advanced interaction and efficient communication, logistical support, workload relief, and ongoing professional development. Conclusions: AI tools such as automated alert systems, predictive algorithms, and decision support transform nursing by increasing efficiency, accuracy, and patient-centered care, improving communication, reducing errors, and enabling earlier interventions with safer and more efficient quality care.

## 1. Introduction

Over time, the evolution of quality and safety in nursing care has been significant; hence, there is great concern for and focus on improving healthcare services [1,2]. Several factors have contributed to these advances, including client involvement, professional and organizational engagement, and the development of client-centered care standards, with an emphasis on safety and evidence-based practice [3,4]. These trends have shaped and enhanced nursing practice globally, influencing the conception and implementation of care.

The organization of nursing care is fundamental to guaranteeing positive results for patients. The Portuguese Order of Nurses, in line with international guidelines, believes that the organization of nursing care involves nurses adopting methodologies for organizing nursing care [5]. These methodologies are aimed at designing, planning, implementing and evaluating the nursing care to be provided to patients according to their needs. It is also important to highlight the implementation of standardized practices, the appropriate allocation of material and human resources, performance monitoring, effective communication, risk management, investment in professional training, continuous training and the use of technological resources as fundamental to care management [6]. These factors can differentiate the provision of high-quality care.

The increasing demand for healthcare services, associated with more complex care requirements, drives the search for innovative solutions to optimize nursing practices [7]. In this context, Artificial Intelligence (AI) has emerged as a disruptive and promising force, offering various tools and techniques that support clinical practice and decision-making. The integration of AI has the potential to bring substantial changes in how nursing care is organized, managed, and delivered [8,9].

The integration of AI into the workflow and work organization of nurses significantly contributes to improving the quality of care, efficiency, and effectiveness of healthcare institutions, resulting in increased satisfaction for both patient and nursing staff satisfaction [10]. In this context, AI can be classified into three domains: clinical, remote care, and care management. In the clinical domain, AI can support decision-making through real-time patient monitoring and automated triage, in addition to improving safety by preventing errors and assessing risks [11]. In the domain of remote care, AI enables remote monitoring and support for teleconsultations, as well as automated interventions in emotional support and mental health for both professionals and patients [12]. In the care management domain, AI optimizes resource allocation based on workload and allows for the automation of administrative tasks [13].

However, it is crucial to address the challenges arising from the implementation of this technology, including ethical issues and the need for adequate training to maximize benefits and minimize risks [14].

Given the importance of this topic and the emerging need to rethink new strategies for the conception, implementation, and organization of nursing care, we identified that there are no systematic reviews or scoping reviews conducted or ongoing on this topic. Thus, we highlight that the organization of nursing care has gained prominence in recent years, which aligns with the growing concern for improving healthcare quality and the need for better health outcomes [15,16]. The lack of systematic evidence on the integration of AI into the organization of nursing care and the potential benefits that could support safer and more patient-centered nursing practice motivated the conduct of this study.

Based on these premises, it is understood that mapping the available evidence on how AI can contribute to the organization and enhancement of nursing care is crucial. Additionally, filling this gap could provide valuable insights for clinical practice and health policy formulation. Thus, the aim is to synthesize the evidence on the use of artificial intelligence in the organization of nursing care.

## 2. Materials and Methods

This review was conducted following the methodology proposed by the Joanna Briggs Institute for scoping reviews, and its writing adhered to the Preferred Reporting Items for Systematic Reviews and Meta-Analyses for Scoping Reviews (PRISMA-ScR) guidelines [17,18]. The protocol for this review was developed and is registered with the Open Science Framework (https://doi.org/10.17605/OSF.IO/VX6TD (accessed on 1 April 2024).

We used the Population, Concept, and Context (PCC) framework to construct the research question: What is the contribution of artificial intelligence to the organization of nursing care? Hence, we emphasize that the PCC framework was followed for defining the eligibility criteria in accordance with the guidelines recommended by the Joanna Briggs Institute [17].

In terms of the population, we considered primary articles that featured nursing professionals as the population, regardless of their category.

Regarding the concept, we included studies that addressed the concepts of artificial intelligence, the organization of nursing care and their relationship. Artificial intelligence is an innovative technology that enables computers to perform tasks requiring intelligence and critical thinking identical to humans [19]. In the healthcare domain, AI can be divided into two categories, namely virtual and physical. Virtual AI includes applications such as electronic health record systems and neural network-based guidance for treatment decisions, while physical AI involves the use of robots assisting in surgeries and smart prosthetics for individuals with disabilities, among others [20].

The organization of nursing care is a multifaceted process that can be executed through one or more work methods. These methods are defined as the infrastructure necessary to facilitate the provision of care to clients in different nursing practice contexts. They depend on the competencies acquired by nurses and the expected outcomes of care delivery. Nurses’ work methods play a crucial role in task allocation and decision-making processes and can reflect aspects such as social values, management ideologies, human resources, or the organizational policies of the institution [21].

Regarding the context, we included studies conducted in any clinical practice context of nurses, as we aimed to conduct a comprehensive review on the topic without specifying contexts.

It is important to highlight that this scoping review will include quantitative studies, experimental, quasi-experimental, prospective, retrospective, case-control, and cross-sectional studies. It will also cover observational studies, qualitative studies such as phenomenological, ethnographic, or descriptive studies, mixed methods studies, reviews, and grey literature. This breadth of studies allows for greater sensitivity in searches aimed at mapping the available evidence.

We followed a three-phase search strategy. Initially, we conducted a limited search in MEDLINE (PubMed) and CINAHL (Cumulative Index to Nursing and Allied Health Literature; EBSCO) to identify articles on the topic under study. By reviewing these studies, we were able to identify terms and keywords to develop a comprehensive and sensitive search strategy. Subsequently, we conducted a second search using the identified keywords and terms in the following databases: MEDLINE (PubMed), CINAHL (EBSCO), Business Source Ultimate (EBSCO), and Scopus^®^. For grey literature, we utilized ProQuest—Dissertations and Theses. The search was conducted in January 2024 using the terms “Artificial Intelligence,” “Nurse Administrators,” and “Nurse Manager,” along with their related terms (Table 1).

We used MeSH terms and CINAHL headings to determine the search terms across several databases. We considered both published literature and grey literature in any language and without a time limit. During the third phase of the search, we reviewed the reference lists of included studies but did not find any additional relevant studies.

After conducting the search, we uploaded all identified studies to the Rayyan platform, and duplicates were identified and removed. Two independent reviewers assessed the titles and abstracts to determine eligibility criteria and conducted a pilot screening of the first 25 titles and abstracts to ensure that the previously defined eligibility criteria were sensitive to the desired outcomes and that consensus was reached among the reviewers. The full text of all studies that met or potentially met the eligibility criteria was analyzed by two independent reviewers. Any discrepancies between the reviewers were resolved through constructive discussion, without the need to involve a third reviewer.

Data extraction was independently conducted by two reviewers who analyzed the full text and recorded all reasons for the exclusion of studies. A data extraction table was developed by the authors for data extraction. The extracted information included details about the studies such as authors, year of publication, country of origin, objective and study type, and the main results that addressed the research question and objective. After data extraction, we performed a thematic analysis of the findings, followed by a narrative synthesis of the main findings.

## 3. Results

**Figure 1:**
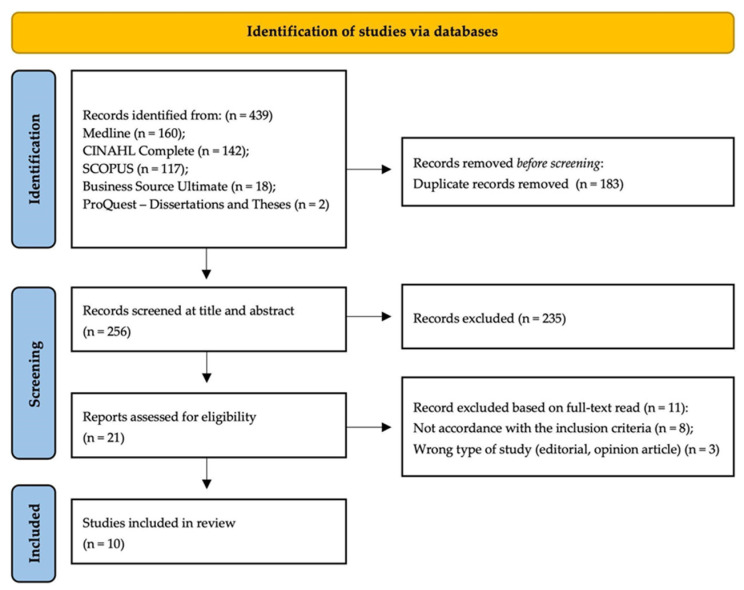
PRISMA flow diagram.

We identified a total of 439 studies across five databases, and 256 studies remained after removing duplicates. After screening the titles and abstracts, 235 studies were excluded. The full text of 21 studies was then read and assessed for compliance with the predefined eligibility criteria. Upon completing all phases of analysis and evaluation, 10 studies were included in this review. The results of the search conducted, as well as the reasons for study exclusion, are presented in the PRISMA flow diagram (Figure 1) [22].

The studies included in the review were published between 2008 and 2022. The countries of origin included China [23,24,25], Colombia [26], Spain [27], the United States of America [28,29,30], Finland [31], and Turkey [32] (Table 2).

The results were synthesized and organized into two themes, namely tools for the organization of nursing care using artificial intelligence, and contributions of using tools for the organization of nursing care using artificial intelligence.

### 3.1. Tools for the Organisation of Nursing Care Using Artificial Intelligence

Within the organization of nursing care, a variety of AI tools are increasingly being used to improve the efficiency, quality, and safety of nursing care. By mapping the articles of this review, numerous AI-mediated tools used in nursing care organization were identified, which were then divided into three models of tools, namely monitoring and prediction, decision support, and interaction and communication technologies.

The monitoring and prediction tools for nursing care included automated alert systems using residential sensor data for health assessment [23,27,31]; machine learning models to predict the development of pressure injuries based on nursing assessment phenotypes [31]; predictive algorithms to identify nursing diagnoses [24,26,32]; surveillance systems [28]; fall risk prevention [27]; and the Rothman index [30].

On the other hand, the decision support tools in nursing care included decision support systems for triage management [31]; clinical decision support systems [23,24,27,28]; nursing expert systems [28]; and health information management systems [23,24,27].

Lastly, the interaction and communication technologies tools for nursing care involved automated classification systems for unstructured clinical notes to aid in the organization and analysis of clinical information [31]; voice recognition systems for direct documentation [31]; electronic reminders [29]; mobile app for emergency services [25]; chatbots and virtual assistance [23,24,27]; robots programmed to assist in patient transfer and mobilization [32]; mobile applications; transparent and explainable AI systems [30]; and process automation for patients [26].

Table 3 summarizes all the tools found and their respective descriptions.

The AI-based tools for nursing care organization encompass a variety of innovative resources aimed at improving the efficiency, quality, and safety of care provided. These technologies include automated alerts, machine learning models, decision support, classification systems, and voice recognition, among others. The use of these tools aims to optimize clinical information management, support the decision-making process, prevent risks, and promote a more effective and proactive approach to nursing care delivery.

### 3.2. Contributions of Using Tools for Nursing Care Organization Utilizing Artificial Intelligence

The integration of Artificial Intelligence (AI)-based tools in the organization of nursing care is significantly transforming the practice of nursing, bringing a set of contributions ranging from improved efficiency and quality of care to the promotion of safer and more patient-centered care. These technological innovations are reshaping how nursing professionals interact with data, make decisions, and perform daily tasks, contributing to a more modern and responsive healthcare environment.

Amongst the contributions, operational efficiency in nursing care [23,24,25,26,28,29,31,32] is significantly enhanced with the use of AI tools. Automated alert systems [24,28] and predictive algorithms [26,32] allow real-time monitoring of patients’ health using data from residential and hospital sensors. These systems can identify patterns and anomalies that might go unnoticed in the overloaded day-to-day workload of healthcare professionals. The ability to predict adverse events [24,29,30,31] is considered one of the main advantages, as it enables early and preventive interventions, reducing complications and improving clinical outcomes.

Another contribution involves decision support [23,24,25,28,29,31,32], with AI tools involving clinical decision support systems in nursing and providing a solid foundation for informed decision-making. These systems use advanced algorithms and evidence-based data to analyze clinical information and offer precise and personalized recommendations [23,24,28,29,30,31]. Hence, they help overcome variability in clinical practice, ensuring that the care provided is consistent and of high quality. Moreover, by alleviating part of the cognitive load on nursing professionals, these tools grant more time to be devoted to direct patient care, strengthening the nurse–patient relationship, and improving patient satisfaction [23,24,27,28,29].

Advanced interaction [29] and communication is another contribution to the use of AI tools, as chatbots and voice recognition systems [23] facilitate efficient and accurate communication both among healthcare professionals and between professionals and patients. These technologies allow direct documentation and quick access to clinical information [28,31], optimization of workflow [26,27] and minimization of errors [23,24,25,26,28,29,30,31]. Additionally, the introduction of mobile apps facilitates nursing professionals to access real-time information and updates [25,28,31], serving as a crucial bridge between the patient’s needs and the caregiver’s actions in dynamic environments.

Logistical support [32] and workload relief [26,27] stand out as contributions of using AI tools in the context of nursing care, as robots programmed to perform non-clinical tasks, such as patient mobility and heavy load transportation, are reconfiguring the work routine of nursing professionals. By taking on these physical and repetitive tasks, robots help reduce the risk of occupational injuries among healthcare professionals and increase their productivity. This allows nursing professionals to focus their efforts and skills on more complex and patient-centered activities, raising the overall standard of care provided [32].

Finally, it is evident that the technological evolution driven by AI also offers numerous opportunities for continuous professional development. Thus, training and education focused on AI are becoming essential to prepare nursing professionals for the future demands of healthcare. By learning to use AI tools effectively, healthcare professionals can expand their competencies and stay updated with evidence-based practices. Continuous development is crucial to ensure that technological innovations are integrated ethically and effectively into daily practice, promoting patient-centered care that respects the individuality and dignity of each person [23,28].

## 4. Discussion

The studies included in this review demonstrate that artificial intelligence has contributed to the organization of nursing care, aiming to improve the efficiency, quality, and safety of the care provided [9,33]. Furthermore, it is noted that AI is not viewed as a replacement but rather as a complement to nursing practice [34].

Artificial intelligence plays a crucial role in monitoring and predicting nursing care, both in hospital environments and in home settings. Its application is particularly significant in data collection and the continuous monitoring of health conditions over time, allowing for the early identification of signs that indicate either deterioration or improvement in a patient’s condition. Furthermore, the insights generated by AI facilitate more precise care, helping to reduce adverse events and directly enhancing the overall quality of care provided [35,36,37,38,39]. The use of AI has demonstrated a positive impact, particularly in enhancing diagnostic accuracy, promoting client-centered care, and reducing adverse events [37,40,41].

The AI developed to support decision-making in nursing care has proven to be a valuable resource. The results indicate the use of algorithms for analyzing data recorded by nurses during patient care, allowing for the processing of large volumes of clinical information, such as medical history, test results, and vital signs. Thus, it is evident that AI can play a significant role in enhancing critical thinking and clinical decision-making [34]. The data provided by healthcare professionals facilitate the assessment and management of patient conditions, allowing for the identification of diagnoses, personalization of care plans, and prioritization of interventions, thus increasing the efficiency of care provided. In this context, AI is crucial for real-time decision-making in challenging health situations, where patient conditions may be more unstable, making it a client-centered strategy and an essential facilitator for clinical practice [42].

Other types of AI have significantly contributed to the management and continuity of patient care, enhancing communication between nurses and the entire healthcare team, as well as optimizing material and human resources, especially in the execution of administrative tasks. The main AI tools used in the management process of nursing work include virtual assistants, chatbots, clinical decision support systems, telemedicine platforms, remote monitoring devices, big data analysis, and augmented and virtual reality technologies [43,44]. Similarly, mobile applications have provided quick access to relevant data, aiding in the organization, delivery, and transition of care [45]. These, AI tools facilitate clinical documentation and enhance communication among healthcare professionals, contributing to safer and more integrated care [46,47].

In this context, AI significantly impacts the nurses’ work, not only by facilitating activity management and time availability but also by reducing workload, allowing greater focus on direct patient care by identifying actual needs and improving care quality [48,49]. It is important to emphasize that, for nursing managers, AI can help organize teamwork, improving effectiveness and professional performance. Additionally, the introduction of robots for non-professional tasks, such as transporting heavy objects, can increase the productivity of the nursing staff [34].

Despite technological advances and improvements in nursing practice, the implementation of AI in nursing care organizations still faces significant barriers. A notable aspect is the lack of clarity on how these technologies can be integrated efficiently and safely, which emphasizes the importance of nurse training, focusing on their professional and continuous development and covering topics such as the use of AI and awareness of its clinical applicability. Thus, the need for workplace training to familiarize nurses with tools available to nursing care organization becomes essential [50]. Likewise, the need for investment in professional training regarding new AI-based tools is essential to ensure they also benefit patients [51].

It is crucial to highlight a fundamental aspect related to the ethical and safe use of AI, safeguarding each client’s integrity and individuality. Ensuring privacy, integrity, and individuality and avoiding potential algorithmic biases or discrimination are essential when using AI [52]. Healthcare organizations must invest not only in adopting these technologies but also in creating an environment that promotes innovation and the acceptance of AI use by healthcare professionals [53]. This involves developing clear policies, fostering a culture of continuous learning, and ensuring all team members understand AI’s benefits and limitations. By investing in such initiatives, it is possible to maximize potential gains in efficiency and quality in nursing care delivery [54].

Despite the transformative potential of AI in nursing practice, which spans from clinical decision-making to work organization and the promotion of patient-centered care, as evidenced by the results of this study, a limitation to consider is that the included studies focus on the use of AI in specific contexts, without clearly demonstrating its contribution to the broader organization of nursing care.

## 5. Conclusions

The increasing integration of AI into nursing care organizations has demonstrated a significant impact on the efficiency, effectiveness, and quality of services provided. These technologies should be seen as complementary to the clinical practice of nurses, as they facilitate quick decision-making, especially in contexts where patients’ clinical conditions are challenging. Additionally, AI enables the implementation of a more patient-centered approach, promoting a more efficient allocation of resources and contributing to the safety of care.

The use of AI also supports timely access to relevant data, assisting nurses in organizing and delivering care, which makes nursing practice more agile and effective. The responsible and secure integration of AI into nursing practice has the potential to positively transform the way care is delivered, from clinical decision-making to work organization and the promotion of patient-centered care. However, the use of AI must be conducted with caution, always safeguarding patient privacy and security through ethical and responsible use of technology.

In summary, AI represents a valuable contribution to the organization of nursing care and should be used as a complement to nursing practice to support quick and accurate decision-making, especially in complex clinical situations.

## Figures and Tables

**Table 1:** Search strategy in databases.

Database (Host)	Search String	Results
Medline (PubMed)	(“Artificial Intelligence”[MeSH Terms] OR “Artificial Intelligence”[Title/Abstract] OR “Deep learning”[Title/Abstract] OR “Machine learning”[Title/Abstract] OR “Information technology”[Title/Abstract]) AND (“Nurse Administrators”[MeSH Terms] OR “nurse administrator*”[Title/Abstract] OR “nurse manager*”[Title/Abstract] OR “nurse executive*”[Title/Abstract] OR “nursing management”[Title/Abstract] OR “nurse management”[Title/Abstract])	160
SCOPUS^®^	ABS (“Artificial Intelligence” OR “Artificial Intelligence” OR “Deep learning” OR “Machine learning” OR “Information technology”) AND ABS (“Nurse Administrators” OR “nurse administrator*” OR “nurse manager*” OR “nurse executive*” OR “nursing management” OR “nurse management”)	117
CINAHL (EBSCO)	AB (MH “Artificial Intelligence” OR “Artificial Intelligence” OR “Deep learning” OR “Machine learning” OR “Information technology”) AND AB (AB (MH “Nurse Administrators” OR MH “Nurse Managers” OR MH “Nursing Management” OR MH “Nurse Executives” OR “nurse administrator*” OR “nurse manager*” OR “nurse executive*” OR “nursing management” OR “nurse management”)	142
Business Source Ultimate (EBSCO)	(AB (DE “ARTIFICIAL intelligence” OR “Artificial Intelligence” OR “Deep learning” OR “Machine learning” OR “Information technology”)) AND (AB (“nurse administrator*” OR “nurse manager*” OR “nurse executive*” OR “nursing management” OR “nurse management”))	18
ProQuest—Dissertations and Theses	“Artificial Intelligence” AND “nurse administrator”	2

* = truncation.

**Table 2:** Characterization of included studies.

Study/Year/Country	Objective	Type of Study	Contributions
Li et al., 2022 [23] China	Investigate the influence of leaders’ innovation expectation on nurses’ innovation behavior in conjunction with artificial intelligence, as well as explore the chain mediating effect of job control and creative self-efficacy between leaders’ innovation expectation and nurses’ innovation behavior.	Cross-sectional survey	Leaders’ innovation expectation helps to enhance nurses’ creative self-efficacy and job control, thereby enhancing nurses’ enthusiasm for innovation.
Chang et al., 2021 [24] China	Investigate how the use of robots can impact nurses’ engagement in professional tasks and reduce engagement in non-professional tasks, as well as analyze how these changes may influence job satisfaction, health perception, and nurses’ intention to turnover.	Cross-sectional survey	The use of robots reduces nurses’ engagement in non-professional tasks and increases their focus on professional tasks, which is positively related to overall job satisfaction and the perception of improved health among nurses.
Dong et al., 2021 [25] China	Develop an emergency nursing management system based on visual artificial intelligence, which aims to enhance clinical work efficiency and information management in hospital emergency environments.	Methodological	The emergency nursing management system based on visual artificial intelligence, once developed and tested, proved to be operational and capable of providing significant convenience for clinical work. The system enhances nurses’ efficiency by facilitating access to and management of medical information, such as patient data and medical orders.
Moreno-Fergusso et al., 2021 [26] Colombia	Provide tools to improve the key performance indicators of inpatient care management, including nurses’ workload, using AI.	Methodological	There are several processes inherent in compassionate nursing care that can be improved using technology. The proposed model presents an opportunity to make almost perfectly balanced nurse-to-patient assignments according to the number of patients and their health conditions using technology.
Ladios-Martin et al., 2022 [27] Spain	Create a model that detects the population at risk of falls by considering a fall prevention variable and assess the impact of this variable on the model’s performance.	Methodological	The demonstration that the inclusion of the fall prevention variable in a machine learning model significantly improves the ability to identify patients at risk of falls in hospital settings.
Courtney et al., 2008 [28] United States of America	Explore how the Nursing Practice Framework, from Novice to Expert, can shed light on the challenges and opportunities in implementing information technology such as clinical decision support systems in nursing practice.	Literature Review	Identification and analysis of the challenges and opportunities in the implementation of Clinical Decision Support Systems in nursing, with a particular focus on the application of the framework. Furthermore, these elements enable shaping the way decision support systems can be developed and implemented in nursing practice, aiming to enhance the quality of patient care.
Piscotty et al., 2015 [29] United States of America	Report the results of a study investigating the relationship between the use of electronic nursing care reminders and the occurrence of missed nursing care.	Cross-sectional survey	The frequent use of electronic nursing care reminders is associated with a reduction in reports of missed nursing care.
Roberty, 2019 [30] United States of America	Explore how artificial intelligence is transforming nursing practice, highlighting the tools and algorithms being used to enhance the delivery of healthcare services.	Literature Review	Artificial intelligence can transform nursing practice by enhancing the quality of care, emphasizing the importance of ethics and transparency in AI systems, and preparing nurses to critically and knowledgeably integrate these technologies into their clinical practice.
Gerich et al., 2022 [31] Finland	To synthesize currently available state-of-the-art research in artificial intelligence-based technologies applied in nursing practice.	Scoping review	Education on nurse informatics for all nursing professionals and students is imperative, and basic knowledge of AI-based technologies in nursing should be incorporated on all professional levels.
Ergin et al., 2022 [32] Turkey	Investigate the perceptions and opinions of nursing managers regarding the use of artificial intelligence and nurse robots in the context of healthcare.	Cross-sectional descriptive	Most nursing managers believe that artificial intelligence and robots can benefit the nursing profession by helping to reduce the workload of nurses, but they do not replace nursing professionals.

**Table 3:** Tools for nursing care organization using artificial intelligence.

Tools	Description of the Tool
**Monitoring and prediction tools for nursing care**	
Automated alert systems using residential sensor data for health assessment [23,27,31]	A tool that enables continuous monitoring of patients’ health outside the hospital environment, utilizing connected devices to collect real-time health data and alert healthcare professionals about potential issues.
Machine learning models to predict the development of pressure injuries [31]	A tool that utilizes algorithms and computational techniques to analyze and identify patterns in patient data, including nursing assessment phenotype information. Based on these data, the model is trained to recognize correlations and predict the likelihood of a patient developing a pressure injury within a specific timeframe.
Predictive algorithms to identify nursing diagnoses [24,26,32]	A tool that utilizes complex algorithms integrated into logical sequences of software to analyze and process various types of medical data. These models can predict potential future scenarios based on the collected information and subsequently facilitate decision-making and actions for the nursing team.
Surveillance systems [28]	Tools to monitor and detect specific events or patterns in real-time. These systems are designed to collect, analyze, and interpret data continuously to identify potential issues or trends that require immediate attention.
Fall risk prevention [27]	A tool that comprises a set of criteria and indicators to assist healthcare professionals in identifying patients at higher risk of falling and implementing appropriate preventive measures.
Rothman Index [30]	A tool used to assess patient severity and the risk of clinical deterioration. This tool is based on data collected from the patient’s electronic medical record and artificial intelligence algorithms.
**Decision support tools in nursing care**	
Decision support systems for triage management [31]	A tool designed to automatically extract and categorize relevant information from unstructured clinical notes, classifying them into different categories or topics. Through natural language processing algorithms, the system can identify linguistic patterns, clinical terms, and specific contexts to intelligently interpret the content of notes.
Clinical decision support systems [23,24,27,28]	A tool that provides healthcare professionals with information and recommendations to assist in clinical decision-making. It relies on algorithms and predefined rules to analyze clinical data, scientific evidence, and patient information to offer personalized, evidence-based guidance.
Nursing expert systems [28]	Tools developed based on rules and algorithms that reflect the knowledge of nursing experts. Expert systems can analyze clinical data, patient symptoms, medical history, and other relevant information to generate personalized, evidence-based recommendations.
Health information management systems [23,24,27]	Tools to collect, store, manage, and transmit information related to patients, healthcare services, and clinical and administrative operations of a healthcare institution. These systems play a crucial role in organizing and enhancing healthcare by enabling quick and secure access to relevant clinical and administrative data.
**Interaction and communication technologies tools for nursing care**	
Automated classification systems for clinical notes [31]	A tool that utilizes natural language processing and machine learning techniques to organize and analyze information in unstructured clinical notes. Unstructured clinical notes consist of free-text narratives written by healthcare professionals during patient care, containing a variety of vital clinical information such as symptoms, diagnoses, treatments, and medical history.
Voice recognition systems for direct documentation [31]	Tools that enable healthcare professionals to record clinical information efficiently and accurately through speech. These systems use voice recognition technology to convert healthcare professionals’ speech into written text, which is then directly incorporated into the patient’s electronic health record.
Electronic reminders [29]	A tool used in nursing care organizations to assist healthcare professionals in remembering important tasks, specific procedures, or relevant information during patient care. These reminders are integrated into health information systems and can be triggered at strategic moments to ensure the proper execution of nursing activities.
Mobile app for emergency services [25]	A tool that utilizes algorithms and artificial intelligence techniques to optimize emergency care and patient triage. These systems are designed to assist healthcare professionals in prioritizing and efficiently managing emergency cases, ensuring that patients receive appropriate treatment promptly.
Chatbots and virtual assistance [23,24,27]	A tool designed to interact with users in a specific manner such as human conversation, providing information, assistance, and support across various contexts.
Robots programmed to assist in patient transfer and mobilization [32]	Tool programmed with algorithms and technologies that enable interaction with patients and perform specific tasks, such as assisting patients in moving them from a bed to a chair, aiding in patient transfer to stretchers or medical equipment, and supporting healthcare professionals during mobilization procedures.
Mobile applications [30]	A digital tool developed for mobile devices, such as smartphones and tablets, aimed at enhancing healthcare delivery and facilitating communication between healthcare professionals and patients.
Transparent and explainable AI systems [30]	A tool created to provide clear and understandable information on how to come to certain conclusions or make recommendations.

## Data Availability

Not applicable.

## Public Involvement Statement

There was no public participation in any aspect of this research.

## Guidelines and Standards Statement

This manuscript was written according to the Joanna Briggs Institute methodology for scoping review research.
